# Supplementary material for: Are carbon emissions trading and green financial instruments synergistic? -Comprehensive quantitative research based on content analysis
Source: PLoS One. 2024 Mar 7;19(3):e0298601. doi: 10.1371/journal.pone.0298601 (PMC10919601; doi:10.1371/journal.pone.0298601)
Supplement: S1 Data — (ZIP) [file pone.0298601.s001.zip › Data/地级市Z_F工作报告.zip!/╔╜╢1⁄2/╟α╡║╩╨/└┤╫╘╣1⁄2╓┌║┼í╛┬φ┐╦╩2╛▌═°í┐_2019.docx]

各位代表：

　　现在，我代表市人民政府向大会报告工作，请予审议，并请各位政协委员和其他列席人员提出意见。

　　一、2018年工作回顾

　　2018年,是青岛历史上极不平凡的一年。一年来，在中共青岛市委的坚强领导下，全市上下坚持以习近平新时代中国特色社会主义思想为指导，深入学习贯彻党的十九大和十九届二中、三中全会精神，认真贯彻落实习近平总书记视察山东、视察青岛重要讲话、重要指示批示精神，坚持稳中求进工作总基调，贯彻新发展理念，落实高质量发展要求，以新旧动能转换重大工程为引领，统筹推进稳增长、促改革、调结构、惠民生、防风险，解放思想，真抓实干，经济社会保持持续健康发展。预计,全市生产总值实现1.2万亿元左右，同比增长7.5%左右；一般公共预算收入达到1231.9亿元，增长6.5%；全体居民人均可支配收入增长8%以上；城镇登记失业率2.94%；居民消费价格上涨2.1%；节能减排降碳任务全面完成。主要做了以下工作：

　　（一）团结一心众志成城，峰会服务保障任务圆满完成

　　上海合作组织成员国元首理事会第十八次会议在我市召开，做好服务保障工作，是习近平总书记和以习近平同志为核心的党中央交给青岛的一项重大政治任务。全市上下坚决贯彻落实习近平总书记重要指示精神，有力有序、精心细致做好场馆改造提升、会务礼宾服务、交通安全保卫工作，同步实施城市环境提升行动，圆满实现了“世界水准、中国气派、山东风格、青岛特色”的服务保障目标，习近平总书记作出重要指示给予充分肯定，与会各方和国内外给予广泛赞誉。期间，全市干部职工勇于担当、攻坚克难、连续奋战，广大市民全力支持、热情参与、无私奉献，展现了良好精神风貌。上合组织青岛峰会的成功举办，给我们带来了千载难逢的发展机遇，不仅极大提升了青岛在国内外的知名度、美誉度和影响力，也给我们这座城市留下了弥足珍贵的物质财富和精神财富，为推动青岛率先走在前列注入了强大动力，开启了全面建设现代化国际大都市新的征程。

　　（二）稳增长转动能，经济发展质量稳步提升

　　着力稳定经济运行。面对下行压力，全面落实减税降费政策，当年为企业新增减负147亿元；出台支持高质量发展政策，加强调度，真抓实干，保持了经济稳中有进良好态势。第二产业增加值增长7.7%左右，第三产业增加值增长7.4%左右。固定资产投资增长7.9%左右，200个市级重点项目完成投资1600亿元。社会消费品零售总额增长9.8%左右，限上批发零售住宿餐饮业营业额分别增长25.4%、11.7%、12.4%、21.8%。接待游客突破1亿人次，增长13%以上。存贷款余额双超1.6万亿元。新登记市场主体28.9万户，增长15%；市场主体总数达到133.9万户，其中企业49.2万户。

　　着力推进新旧动能转换重大工程。发布实施意见和规划，确定优先发展的“956”重点产业集群。设立55支新旧动能转换基金，推进970个项目建设，完成投资1800亿元。推进“双百千”行动和“一业一策”计划，本地营业收入过千亿元行业6个、过百亿元行业43个，过千亿元企业1家、过百亿元企业28家。生物医药、节能环保、商务服务等产业增加值均增长10%以上，大数据、云计算产业营业收入增长20%以上。抓好400个工业转型升级项目，两个投资百亿级集成电路项目加快推进，一汽大众、上汽通用五菱和北汽新能源汽车项目加快建设。董家口、新河化工产业园获省政府认定。海尔工业互联网平台、海信智能家居成为国家级示范平台。成为首批5G试点城市。

　　着力增强自主创新能力。坚持梯次培育，高新技术企业超过2700家，国家科技型中小企业1812家，分别占全省的30%、25%左右。杰华生物成为全省首家独角兽企业。高技术产业增加值增长7%左右。坚持集聚建设，国家重点实验室达到9家，省级以上企业技术中心、工程（技术）研究中心分别达到193家、166家。海洋科学与技术试点国家实验室深海观测技术实现突破。中科院海洋大科学研究中心、国家高速列车技术创新中心加快建设。坚持成果导向，有效发明专利拥有量增长20.2%，有效注册商标增长31.9%。技术交易额增长23%。14个项目获国家科学技术奖。坚持招才引智，新增人才21.9万人，其中，新增聘任院士4人，新增国家级、省级、市级各类人才工程人选453人，人才总量达到193万人。

　　（三）勇于担当服务大局，重大战略实施全面起势

　　深入落实海洋强国战略。大力发展海洋经济，国际海洋名城加快建设。海洋生产总值增长15%左右，占全市生产总值26.5%左右。海洋化工、海洋工程建筑业增加值均增长20%以上。港口新增集装箱航线13条，货物和集装箱吞吐量分别达到5.4亿吨、1931.5万标箱。

　　深入落实军民融合发展战略。积极探索军民平台融合、领域融合、区域融合发展模式，加快建立海军、海洋、海防融合发展机制。军民融合项目加快推进，哈工程船舶科技园17个项目加快建设。成功举办首届军民融合科技创新成果展、全国标准化军民融合年会。

　　深入落实乡村振兴战略。20个乡村振兴工作队深入基层开展工作。建成986个现代农业园区，农业适度规模经营比重达到67%，新型农业经营主体达到2.5万家。粮食总产300万吨，水产品总产118万吨。“三品一标”农产品1042个。成功创建国家农产品质量安全市。农产品出口额50亿美元，居全国城市第一位。完成398公里农村公路改建整修、1649个农村电网改造项目、165个村庄饮水安全设施提升工程。建设美丽乡村示范村100个、达标村1000个，省级美丽乡村达标村达到63.7%。

　　（四）深化改革扩大开放，发展动力城市活力与日俱增

　　改革开放40年，青岛始终与时代同步、与祖国同行，发生了翻天覆地的变化，取得了令人瞩目的成就，涌现出一批勇于探索的先进人物，创造了一批影响全国的改革样本。许振超、张瑞敏、周明金3位同志被授予“改革先锋”称号，8人（群体）和7件事分别入选全省改革开放40周年感动山东人物和最具影响力事件。他们是这座城市的骄傲。

　　重点领域改革取得新成效。争取国家、省改革试点任务59项。大力推进一流营商环境提升年行动，市级行政审批事项和权力事项分别精简62.6%和55.3%，“一次办好”基本实现全覆盖，市级审批事项网上办理率达到94%，市行政审批服务大厅全面实行“一窗受理”。口岸整体通关时间压缩45%，集装箱进出口合规成本降低14%。国资国企改革加快推进，市直企业资产、主营业务收入分别增长15.3%、11.3%。民营经济稳步发展，实现税收、就业分别占全市的51%、76.8%。金融综合改革不断深化。青岛财富产品交易中心投入运营。新引进11家金融机构。全省通过证监会A股发行审核的6家公司均来自青岛，海尔成为首家在中欧所上市的中国公司。三板、四板新挂牌企业450家，蓝海股权交易中心挂牌企业达到1529家。银行不良贷款率降至1.87%，低于全国、全省平均水平。户籍制度改革取得新成效，人才落户、居住落户、项目落户、积分落户更加便捷。全部完成6015个村集体资产清产核资任务,完成5550个村集体产权制度改革。

　　对外开放取得新突破。国际城市战略深入实施，我市做法获国务院通报表扬。开展“千企招商大走访”，走访企业上万家，签约项目661个。赴日本、韩国、德国、美国等国家和北京、上海、深圳、香港等城市举办招商推介会。实际利用外资86.9亿美元，增长10%，占全省43%。实际到位内资2156亿元，增长14.2%。国际贸易总额实现1270亿美元，增长10%；其中，货物进出口5321亿元，增长5.7%。跨境电商交易额增长41.9%。服务外包合同额增长39.7%，居全国服务外包示范城市第8位。中国—上合组织地方经贸合作示范区总体建设方案上报国务院，多式联运中心正式启用。深度融入共建“一带一路”，对沿线国家地区投资额、进出口额达到6.8亿美元、2590亿元，占全市的26%、48.7%。保税港区全域封关，国家汽车平行进口试点启动实施。获批外国人144小时过境免签政策。新增3个国际友城、20个国际经济合作伙伴城市。成功举办国际渔业博览会、东亚海洋合作平台青岛论坛、世界旅游城市联合会峰会、上合组织国家电影节、上合组织国家进口商品展、日韩进口商品展等重要国际性活动。青岛国际啤酒节参节人数创纪录达到630万人次。流亭机场新增航线37条,旅客吞吐量达到2454万人次。火车站发送旅客达到3454万人次。

　　（五）加快建设时尚美丽青岛，城市环境品质显著提升

　　深入开展美丽青岛三年行动，完善基础设施，提升市容市貌。整治建筑立面9704栋，亮化提升一批建筑、路段和景点，浮山湾夜景灯光秀成为城市新名片。拆除违法建设844万平方米，完成楼院整治500个，新建改建绿地799公顷，建设口袋公园189个。新建改建公厕392座，实施农村改厕89200座。通过国家卫生城市复审测评。济青高铁、青盐铁路、董家口疏港铁路开通运营，青岛西站投入使用，红岛站基本建成，胶东机场主体竣工。轨道交通在建155公里，11号线、13号线开通，运营线路达到4条共计172公里，全年运送乘客1.55亿人次。着力改善市区交通环境，改造220条道路，打通27条未贯通道路，开辟调整107条公交线路，新增公共停车场泊位1.2万个。建成海绵城市131平方公里、地下综合管廊52.5公里。

　　坚决打好污染防治攻坚战。PM2.5、PM10平均浓度分别改善8.1%、5.3%，空气质量优良率达到85%，提高6.4个百分点。城市节能保温改造531万平方米。新增清洁能源供热能力2071万平方米。新增农村清洁取暖46580户。城市公交车新能源、清洁能源使用率达到85%。严格落实河长制、湾长制、湖长制。新建改建2座污水处理厂，整治提升河道245公里，污水处理在全国36个大中城市考核中名列第一。入选全国黑臭水体治理示范城市。完成10座水厂深度处理改造。新增造林13.5万亩，治理水土流失面积69平方公里。扎实推进中央环保督察、国家海洋督察、省环保督察反馈意见整改，解决各类环境问题3000多个。

　　（六）加大投入强化保障，居民生活质量持续改善

　　全市财政用于民生支出1156.2亿元，增长9.3%。10件32项市办实事全部完成。城镇新增就业77.6万人，政策性扶持创业1.9万人。落实新修订的个人所得税法过渡期政策，11月、12月为市民减税5.4亿元。新建改建60所普惠性幼儿园、58所中小学、100所中小学标准化食堂，完成300所农村薄弱学校信息化改造。市立医院东院二期、青大附院东院二期建成使用。增设18个院前急救站。连续14年提高企业退休人员养老金水平。向9.8万城乡低保人员发放低保金7.1亿元。长期护理保险制度惠及1.8万名失能失智人员。14种高值特效药品纳入补充医保目录。实施医疗救助8.19万人,发放救助金2.7亿元。完成住房保障7986套（户），启动棚户区改造41248套（户），完成农村危房改造1200户。推进精准扶贫、精准脱贫，200个省定贫困村、310个市定经济薄弱村、10个市定经济薄弱镇全部摘帽。对口支援和扶贫协作工作成效显著。安全生产形势总体平稳，事故起数、死亡人数进一步下降。食品药品抽检合格率分别达到98%、100%。扫黑除恶专项斗争深入开展，立体化信息化社会治安防控体系不断完善，社会治安环境持续优化。

　　文明城市创建工作成效明显。胶州、平度入选全国新时代文明实践中心试点。邻居节经验在全国得到推广。市民文化艺术节等文化惠民活动蓬勃开展。庆祝首届中国农民丰收节活动成功举办。国家文物局水下遗产保护中心北海基地正式启用。故宫精品文物展成功举办。国家文化消费试点城市工作深入推进。东方影都落成启用，入驻影视及配套企业105家。广泛开展全民健身运动。成功举办第24届省运会、第10届省残运会、第4届省老年运动会、2018青岛马拉松等赛事。青岛代表团在第24届省运会上包揽7个奖项第一。双拥共建持续加强，军政军民关系更加融洽。司法行政、民族宗教、外事、侨务、对台、气象、人防、统计、档案、史志、仲裁等工作得到加强，妇女儿童、青少年、老龄、残疾人、红十字、慈善等事业全面发展。青岛以更加独特的魅力吸引了世人的目光。

　　（七）依法全面履行职能，行政服务水平不断提高

　　认真落实向人大报告、向政协通报制度。向市人大常委会提交地方性法规草案5件，制定政府规章5件、修改32件、废止84件。办理人大代表建议413件、政协提案675件。坚决执行市人大及其常委会的决议、决定，自觉接受市人大法律监督、工作监督和市政协民主监督。扎实推进“大学习、大调研、大改进”和解放思想大讨论，政府工作人员思想境界、作风效能得到新提升。深入开展领导干部联系代表委员、联系企业、联系重点项目和走访基层活动。连续十年举办“向市民报告、听市民意见、请市民评议”活动，网络在线问政、行风在线、政务服务热线等政民互动渠道更加畅通。政府系统全面从严治党不断深化，党风廉政建设不断加强。中央巡视组反馈问题整改扎实推进。充分发挥审计监督作用，政府权力运行更加规范。认真贯彻中央和省委、市委关于激励广大干部新时代新担当新作为的意见精神，干事创业的氛围更加浓厚。

　　各位代表，过去一年，成绩来之不易。这是以习近平同志为核心的党中央坚强领导的结果，是省委省政府和市委科学决策的结果，是市人大、市政协和社会各界监督支持的结果，是全市人民团结拼搏的结果。在此，我代表青岛市人民政府，向全市人民，向全体人大代表和政协委员，向各民主党派、工商联、各人民团体、社会各界人士和离退休老同志，向驻青部队和武警官兵，向中央、省驻青单位，向所有关心支持青岛发展的海内外朋友们，表示崇高的敬意和诚挚的感谢！

　　同时，我们也清醒认识到，当前经济运行稳中有变，经济面临下行压力，实体经济面临一些发展中的困难，民间投资不旺，对外贸易存在不确定性；传统产业转型升级慢，新兴产业比重偏低。民生和社会事业方面还有一些薄弱环节，城乡治理、环境保护等领域还存在一些短板。有些政府工作人员在解放思想、更新观念、担当作为方面还有差距，服务水平有待提升，营商环境仍需优化，政府系统违反中央八项规定精神的问题和腐败现象仍然存在。我们一定正视问题、直面挑战，拿出真招、实招、硬招，切实加以解决。

　　二、2019年经济社会发展总体要求和主要目标

　　各位代表！今年是新中国成立70周年，是全面建成小康社会、实现第一个百年奋斗目标的关键之年。经过改革开放40年的发展，青岛综合实力、竞争力和影响力持续提升，为加快新旧动能转换、实现高质量发展奠定了坚实基础。特别是习近平总书记视察山东、视察青岛重要讲话，对上合组织峰会圆满成功举办作出“办好一次会，搞活一座城”重要指示，为青岛新时代改革发展把航定向。国家布局山东新旧动能转换综合试验区建设，赋予青岛发挥核心引领作用、加快形成国家东部地区转型发展增长极的重任。我们将乘峰会东风，紧紧抓住并持续放大青岛发展重要战略机遇，开拓创新，真抓实干，加快建设现代化国际大都市，谱写青岛社会主义现代化建设的崭新篇章。

　　今年政府工作的总体要求是：以习近平新时代中国特色社会主义思想为指导，认真贯彻党的十九大和十九届二中、三中全会精神，深入落实习近平总书记视察山东、视察青岛重要讲话、重要指示批示精神，牢牢把握“两个走在前列、一个全面开创新局面”总要求，统筹推进“五位一体”总体布局，协调推进“四个全面”战略布局，坚持稳中求进工作总基调，坚持新发展理念，坚持推动高质量发展，坚持以供给侧结构性改革为主线，坚持深化市场化改革、扩大高水平开放，加快建设现代化经济体系，紧紧围绕突出创新引领、实现三个更加目标要求，落实一三三五工作思路，以新旧动能转换重大工程为引领，聚焦聚力经略海洋、军民融合、乡村振兴、打好三大攻坚战、打造对外开放新高地等工作重点，统筹推进稳增长、促改革、调结构、惠民生、防风险工作，保持经济运行在合理区间，进一步稳就业、稳金融、稳外贸、稳外资、稳投资、稳预期，着力增强微观主体活力，加快塑造高质量发展新优势，促进经济持续健康发展和社会和谐稳定，以优异成绩庆祝中华人民共和国成立70周年。

　　主要预期目标是：全市生产总值增长7%左右，一般公共预算收入增长5%以上，社会消费品零售总额增长10%左右，投资稳定增长结构优化，外贸稳增提质，全体居民人均可支配收入增长7.5%左右，城镇登记失业率4%以内，居民消费价格涨幅3%左右，全面完成国家和省下达的节能减排降碳约束性指标和环境质量改善目标。

　　工作中，我们将把握好以下方面：

　　——坚持解放思想争一流。适应新时代发展要求，牢牢把握率先走在前列的目标定位，跳出青岛看青岛、站在未来看现在，进一步拓宽视野、提升境界、更新观念、提高标准，始终保持永不懈怠的精神状态和一往无前的奋斗姿态，以思想大解放引领经济社会大发展。

　　——坚持高质量发展。贯彻落实“巩固、增强、提升、畅通”八字方针，坚持新发展理念，深化供给侧结构性改革，加快推动质量变革、效率变革、动力变革，加快建设现代化经济体系，实现更高质量、更有效率、更加公平、更可持续发展。

　　——坚持稳中求进。正确处理“稳”与“进”的辩证关系，保持战略定力，沉着应对各种风险挑战，深入实施新旧动能转换重大工程，大力发展“四新四化”，积极开展“双招双引”，加速迈向创新驱动、内生发展的快车道，保持经济稳中有进、进中向好。

　　——坚持以人民为中心的发展思想。从解决群众最关心最直接最现实的问题着手，持续做好普惠性、基础性、兜底性民生建设，不断提高城乡居民收入水平，提高公共服务共建能力和共享水平，努力使人民群众获得感、幸福感、安全感更加充实、更有保障、更可持续。

　　三、2019年重点工作安排

　　今年经济社会发展任务十分繁重，要统筹兼顾、优化布局、突出重点，全面抓好各项工作落实。

　　（一）加快建设现代化产业体系。以“双百千”行动和“一业一策”计划为抓手，培育壮大“956”重点产业集群，加快构建创新引领、协同发展的现代化产业体系，打造制造业强市和服务业中心城市。

　　改造提升传统产业。坚持以新技术、新业态、新模式推动传统产业转型升级。引导企业加大技术改造力度，提升装备技术水平。实施智能制造工程，引导企业实施数字化升级改造，推进60个智能互联工厂、数字化车间、自动化生产线示范项目建设。推进制造业、服务业有机融合，推动工业企业向以制造为基础的产品、技术、服务等综合供应商转变。提升食品饮料行业精深加工能力。推动纺织服装行业品牌化、定制化发展。促进化工行业集约、绿色发展。引导传统服务业创新商业模式、服务方式。推动批发企业更大规模聚集、发挥更大辐射作用，鼓励发展新零售。快速提升多层次的住宿接纳能力，积极发展休闲度假主题酒店和特色民宿。大力发展安全放心、高品质有特色的餐饮业，培育为居家烹饪配套的服务企业。

　　培育壮大战略性新兴产业和高技术产业。瞄准新一代信息技术、生物医药、新能源汽车、高端装备、新材料等领域，引龙头、聚配套，规模化集群化发展。加快中电科电子信息装备产业园、中科曙光全球研发总部基地、浪潮大数据产业园、青岛（芯园）半导体产业基地、青岛芯谷等园区建设。实施大数据发展行动，加强新一代人工智能研发应用。促进修正药业、海王生物、国药医疗等项目建设，支持现有药企加快发展。依托整车生产龙头企业，加快新能源汽车产能扩大及配套项目建设。加快推进国家高速列车技术创新中心等重点项目。发展航空产业。培育工业机器人、增材制造、仪器仪表等智能装备产业。推进石墨烯、碳纤维等新材料开发应用。

　　做优做活现代服务业。推动生产性服务业向专业化、高端化发展，生活性服务业向便利化、精细化发展。培育壮大地方法人金融机构，吸引国内外金融机构在青设立分支机构和功能性总部。加快青岛跨境电商产业园、董家口铁路物流园、胶州多式联运中心等项目建设，发展智慧物流、供应链物流。放大港口、机场、高铁等交通设施快速提升的效应，大力发展枢纽经济。积极推动旅游大项目建设，推出个性化、品质化旅游线路和旅游产品，加强网络营销、精准营销，打造国际一流滨海度假旅游胜地。发展文化创意产业，做大影视演艺、动漫游戏、出版发行、数字传媒产业。推进崂山湾国际生态健康城、西海岸新区智慧科技健康城、高新区蓝色生物医药城建设，打造医养健康产业高地。丰富养老服务业态，重视居家养老，加强养老机构建设，促进养老服务连锁化、专业化发展。发展科技服务业。引进培育知名展会，做大会展业规模。发展总部经济、工业设计、会计审计、法律、检验检测服务，打造高端商务中心。

　　加强质量品牌建设。实施质量提升专项行动，发挥大企业示范作用，带动中小企业加强全面质量管理。实施标准引领工程，培育一批国家标准、国际标准，推动标准体系由工业向经济社会各领域覆盖。实施品牌驱动工程，打造青岛制造、青岛服务、青岛购物、青岛文化、青岛旅游五大品牌，加强品牌整体推广和城市综合营销，持续提升“品牌之都”影响力。

　　（二）深入实施创新驱动发展战略。紧紧抓住创新这个“牛鼻子”，大力推进科技创新、商业模式创新、管理创新，引领产业转型升级，实现高质量发展。

　　充分发挥企业的创新主体作用。支持企业广泛开展自主创新和产学研合作，力争有创新活动的规上工业企业占比提高3～5个百分点。支持企业建设创新中心、工业设计中心、工程（技术）研究中心和重点实验室，推进7个省级制造业创新中心建设。大力培育创新型领军企业，力争培育“瞪羚”企业、“隐形冠军”企业20家。实施科技型企业培育“百千万”工程，壮大科技型中小微企业队伍，力争高新技术企业达到3200家。

　　充分发挥各类平台的创新集聚作用。建设好重大科技创新平台，推进中科院海洋大科学研究中心、中科院洁净能源创新研究院等加快建设，支持海洋科学与技术试点国家实验室跨越式发展。积极推动国际院士港等科技创新资源集聚平台更好发挥作用。支持建设一批产业链协同融合、开放共享的公共研发服务平台。推进国家双创示范基地、国家级孵化器等双创平台建设。更好发挥驻青大学、科研机构对企业创新和产业升级的引领和推动作用。支持“蓝色药库”建设。组织20次企业、高校、科研机构对接活动。建立多层次技术转移服务体系，建设国家科技成果转移转化示范区。加强知识产权保护和运用。

　　充分发挥人才的创新支撑作用。突出产业需求，突出“高精尖缺”，持续推进百万人才集聚行动，打造人才高地。深入实施人才支撑新旧动能转换五大工程，引进培育一批产业领军人才和高层次、高技能人才。办好“蓝洽会”“海外院士青岛行”“百所高校千名博士青岛行”等活动。加强海外人才离岸创业创新基地建设。实施大学生在青就业创业计划。落实好大学毕业生先落户后就业、人才安家补贴等人才政策。强化科技成果转化激励，赋予科研人员更大的人财物自主支配权，激发人才创新活力。

　　（三）促进消费快速增长。顺应需求新变化，提升优质产品和服务供给能力，建设面向本地居民和来青游客的消费便捷之城，打造服务周边、面向全国的区域性消费中心城市，争创国际消费中心城市。

　　丰富消费品供给。围绕衣食住行游购医育文教体娱养，进行市场细分行业细化，深度挖掘传统消费不断扩容的潜力，积极拓展新兴消费领域。扩大文化体育消费，升级健康休闲消费，促进养老家政消费，积极培育网络、定制、体验、智能、时尚等消费新热点。各区市立足功能定位和产业基础，确立产业特色、集聚商业品牌、打造消费地标，形成相互补充、错位发展的消费发展格局。

　　加强消费载体建设。启动新一轮商业网点布局规划，推进大型批发市场、商城、购物中心、连锁超市、便利店以及各类餐饮、健身、影院等营业场所建设。结合老城区改造，打造一批新兴消费聚集的商圈、商业街，争取国家高品质步行街试点。引导实体店和网购融合发展。支持发展综合性和专业性网络消费平台。办好城市购物节、中国国际消费电子博览会等交易会，持续培育有影响力的展销、节庆品牌。

　　改善消费环境。优化交通体系，完善区域、城乡和内外贸一体的现代流通网络。加快供应链创新与应用，提高流通标准化、信息化、集约化水平。建设快递示范城市，发展城乡高效配送。提高公共区域免费WiFi覆盖面，加快便捷支付推广应用。加强商品质量、食品药品安全、市场秩序综合监管和治理，健全商品追溯制度和消费者维权机制，营造安全放心的消费市场环境。

　　（四）扎实扩大有效投资。聚焦补短板、调结构、增活力，加快推进总投资6000亿元左右的200个市级重点项目，优化投资结构，提高投资质量，切实发挥投资的关键作用。

　　加强基础设施投资。加快机场、港口、高铁、公路等城市间交通和地铁、市政道路、环卫设施等城市内基础设施建设进度。实现胶东机场转场，加快机场高速公路等配套路网建设。推进潍莱高铁建设。加快地铁1号、4号、8号线建设，实现2号线一期全线贯通，力争开工6号线等新线路。不断优化市政道路设计，加大关键节点设施建设力度。推进智慧城市建设，加大工业互联网、物联网等新型基础设施投资力度，加快5G商用步伐。

　　扩大工业和民间投资。挖掘本地企业潜力，加大招商引资力度，实现工业和民间投资较快增长。推进115个工业重点项目建设，带动配套产业跟进投资。落实市场准入负面清单，健全项目推介机制，完善合理回报，推出一批政府和社会资本合作项目。

　　强化农业农村建设投资。加大农田水利设施建设力度。加强病险水库除险加固、小型水利工程防洪隐患治理工程建设。推进黄水东调二期承接工程建设。加快农村“七改”“四好农村路”建设，促进农村基础设施提档升级。推进农产品食品交易中心等重点项目建设。

　　（五）坚定不移深化市场化改革。坚持向改革要动力，推动重点领域和关键环节改革纵深推进，增强微观主体活力。

　　推进国资国企改革。依法合规赋予企业经营自主权。优化调整国有资本布局，推动资源向优秀企业、优势产业集中。引导企业聚焦主业发展。积极推进混合所有制改革。深化股权激励试点，激发国有企业发展活力。完善国有资产监管体制，强化细化监管措施，加快各级国有企业统一监管。完善国有金融资本管理体制。

　　推进财税和地方金融改革。深化财政事权和支出责任划分改革。推进预算绩效管理改革、政府采购制度改革、政府购买服务改革和涉农资金整合。全面落实国家更大力度的减税降费政策。深化投融资体制改革。支持企业上市融资和发行债券，支持企业在三板、四板市场挂牌交易。争取新增5家以上上市公司。鼓励投资基金以股权、债权、夹层投资等方式灵活多样地为项目和企业提供融资服务。推动财富管理综合改革试验区创新政策落地实施，在跨境财富管理、服务实体经济方面取得新成果。

　　推进改革试点突破。积极争取并全力做好国家、省赋予的改革试点任务。精准对接发展所需、基层所盼、民心所向，加快健康中国示范市试点、平度国家中小城市综合改革试点、全国农村产业融合发展试点、土地二级市场试点、“亩产效益”资源市场化配置试点等112项改革进程，不断完善体制机制，增强经济和社会发展活力。

　　推进政府职能转变。完成市、区（市）政府机构改革，提高政府工作效能和服务水平。大幅减少政府对资源的直接配置，强化事中事后监管，凡是市场能自主调节的就让市场来调节，凡是企业能干的就让企业干。启动公务员分类管理改革，扩大聘任制公务员试点范围。深化功能区职员管理改革。加强镇（街）服务能力建设。

　　（六）坚定不移扩大高水平开放。推进更高层次的对外开放，加快形成全方位深层次立体式开放新格局，打造新时代对外开放新高地，发挥好青岛在全省对外开放中的龙头作用。

　　提高利用外资质量。坚持选准方向、找准对象、用准方法、精准施策，瞄准重点领域重点国家重点外企，扎实推进招商大走访和现代产业精准招商行动，确保利用外资稳定增长结构优化。推动青岛企业境外上市。办好赴日本、韩国、德国等国家招商交流活动。积极对接国际通行经贸规则，保护外商合法权益。

　　促进外贸稳定增长。妥善应对各种不确定性，努力稳定、巩固传统市场，加强与新兴市场的贸易合作。提升国家服务外包示范城市建设水平，扩大服务贸易规模。发展贸易综合服务、跨境电商、公共海外仓等新业态新模式。推进境外经贸合作区建设，带动装备、技术、产品出口。积极扩大进口，建设国家进口贸易促进创新示范区，发展保税展示销售、进口商品直销和进境免税店。优化通关流程，压缩通关时间，加强收费监管。办好上合组织国家投资贸易博览会、日韩进口商品博览会、亚洲农业与食品产业博览会等展会。

　　加强对外经济合作。发挥新亚欧大陆桥经济走廊主要节点城市和海上合作战略支点优势，面向上合组织国家、对接日韩、拓展欧亚、辐射亚太，创建“一带一路”地方经贸合作示范城市。推进多式联运示范工程，加密国际班列，打造欧亚大通道战略枢纽城市。海铁联运、国际班列发送箱量分别增长10%、15%。积极开拓对外承包工程和劳务合作市场。深化与国际友城、国际经济合作伙伴城市的交流合作。加快建设青岛都市圈，强化与周边城市的融合发展，深化与沿黄海、环渤海城市的交流合作。

　　深入实施国际城市战略。落实“国际化+”行动计划，全面推进企业、市场、产业、园区、城市国际化建设。规划建设国际化学校、医院、社区，完善涉外综合服务体系，推进街名路牌、公共标牌和建筑物等外文标识规范化，营造友好便捷的国际人居环境。增开国际航线。复制推广自贸区经验，积极申建山东自由贸易试验区（青岛片区）。办好跨国公司领导人青岛峰会、博鳌亚洲论坛全球健康论坛大会等活动，提升国际影响力。

　　（七）加快建设国际海洋名城。立足海洋优势和特色，坚持科技兴海、产业强海、生态护海，努力在全国海洋经济发展中率先走在前列，当好海洋强国、海洋强省建设生力军。

　　巩固提升传统优势,建设海洋科教名城。加快中船重工海洋装备研究院、国家海洋设备质量检验中心等项目建设，发挥海洋类创新平台带动作用，打造具有国际影响力的海洋科技创新策源地。推进“健康海洋”等重大科研项目攻关，在海洋领域突破一批核心技术。组建国际海洋科技创新联盟。推进国家海洋技术转移中心建设。支持驻青大学涉海学科加快发展。办好国际海洋节。提高海洋科学教育文化国际交流合作水平。

　　大力发展海洋经济,建设海洋产业名城。加快推进100个海洋重点项目建设，力争海洋经济增加值规模和占比持续提升。加快建设海洋牧场、渔港经济区，挖掘远海潜力，推动海洋渔业提质增效。大力提升海洋制造业竞争力，加快发展海洋工程装备、高技术船舶等海洋设备制造业，加快发展海洋生物医药产业、涉海产品及材料制造业。统筹布局海水淡化项目，打造集海水淡化装备、技术、服务于一体的综合供应基地。推动滨海旅游、海洋交通运输业以及其他涉海服务业量质齐升。加大海洋生态环境保护力度，建设绿色可持续的海洋生态环境。深入开展“蓝色海湾”整治行动，建设国家级海洋生态文明示范区。

　　建设一流海洋港口,打造国际航运名城。优化港口集疏运体系，推进董家口港原油码头二期工程、前湾港自动化码头二期工程建设。支持青岛港集团做大做强，加强海运企业引进合作。推动国际邮轮港跨越式发展，深化与邮轮运营商、旅行社合作，快速提升我市在全国邮轮经济中的地位和作用。增开航线，拓展市场，加快提升货客运输量。建设航运大数据综合信息平台，完善国际航运交易、贸易、金融服务功能，建设国际航运中心。

　　促进军民融合发展,建设军民融合创新示范区。强化示范带动，创新体制机制，推动国防建设与经济社会融合发展，加快形成可复制可推广的经验。加大基础设施、创新平台统筹和共建共用力度，加快“军转民”“民参军”步伐，打造军地协同创新体系。积极推进西海岸（古镇口）核心区军民融合产业园建设，壮大军民融合产业规模。积极承接国家军民融合重大示范项目，加快军民融合综合公共服务平台等项目建设。

　　（八）扎实推进乡村振兴战略。坚持农业农村优先发展，推动乡村产业、人才、文化、生态、组织全面振兴,为打造乡村振兴齐鲁样板作出青岛贡献。

　　推动农业全面升级。促进农村一二三产业融合发展,培育市级以上龙头企业300家以上，打造农副产品加工、批发两个千亿级产业集群和乡村旅游、农村电商两个百亿级产业集群。启动建设300万亩粮食生产功能区。全域创建“新六产”示范区，规划建设西海岸高端智慧农业示范区、即墨种都核心区、胶州高效农业先行区、平度绿色粮油终端消费引领区、莱西农产品加工物流先导区。推广标准化生产，培育农产品品牌。发展“互联网+现代农业”，培育农产品电商平台。提升农业科技装备水平，新技术推广应用100万亩以上，农作物生产综合机械化率达到88%。

　　推动农村全面进步。扎实推进农村人居环境整治，实施美丽乡村“十百千”工程，新创建10个农村新型示范社区、100个美丽乡村示范村和1000个达标提升村。加强村庄规划设计，塑造有特色、高品质的村庄风貌。以建制镇为重点，推进污水处理设施建设。规模化畜禽养殖场全部配建粪污处理设施。实施农村冬季清洁取暖改造4.7万户。深化承包地“三权分置”改革。完成95%以上村庄的农村集体产权制度改革。积极发展经济合作社、股份经济合作社等新型农村集体经济组织。

　　推动农民全面发展。推进乡村振兴实用人才培育工程，年内培训1.5万人。实施农村归雁计划，支持高校毕业生、退役军人等返乡就业创业。完善农村文化设施，优化文化产品供给。提高农村社区服务中心规范管理水平，建设“乡村30分钟公共服务圈”。

　　（九）坚决打好三大攻坚战。巩固已有成果，聚焦突出问题，精准施策、扎实推进，加快形成标本兼治、系统发力的良性机制。

　　打好防范化解重大风险攻坚战。强化地方金融监管体系建设，加强企业金融风险排查防控，积极稳妥化解银行不良贷款，提高金融风险防控水平。依法规范政府举债行为，强化债务风险监测和应对，有效管控政府债务。严厉打击非法集资以及其他各类非法金融活动。

　　打好脱贫攻坚战。巩固脱贫成效，提高脱贫质量，着力解决影响“两不愁三保障”的突出问题。完善动态调整机制，对新致贫、返贫群众及时帮扶救助，及时研究、妥善解决各种新问题。持续抓好对口支援和扶贫协作工作。

　　打好污染防治攻坚战。抓好中央环保督察及“回头看”、国家海洋督察、省环保督察交办问题和反馈意见整改落实。加快调整“四个结构”，推进“四减四增”，强化源头治理。推动企业实施清洁生产、超低排放等达标排放改造。突出扬尘、柴油货车等重点污染源治理，打好蓝天保卫战。严格落实河长制、湾长制、湖长制，加强河道综合治理、黑臭水体治理，打好碧水保卫战。加强土壤污染防治，实施农业投入品减量增效和清洁化生产计划，打好净土保卫战。强化危险废物、医疗废物监管，确保安全处置。

　　提升生态文明建设水平。实施“绿满青岛”国土绿化行动，抓好北部绿色生态屏障建设，新增造林13万亩。编制生态保护红线、环境质量底线、资源利用上线和环境准入清单。加强生态保护与修复治理，坚决整治自然保护区突出生态问题。全面推进节能、节水、节地、节材。加快新增建设用地计划“增存挂钩”，建立亩均效益评价制度，有效盘活批而未供、供而未用土地。推行生态环境损害赔偿制度。强化生态环境监测监察执法。加强环境风险防控、应急处置和救援保障能力建设。

　　（十）促进民营经济发展壮大。支持各类市场主体依法合规平等竞争，着力解决制约民营经济发展的问题和困难，为民营经济营造更好发展环境。

　　加强要素保障。启用“金企通”融资服务平台，开展民营企业融资对接专项行动。加大小微企业贷款风险补偿力度。加大对民营企业发展用地支持保障力度。加强企业用工对接，落实稳岗补贴制度。着眼企业需求，开展技能培训，组织专场招聘，加强对口帮扶劳务协作。

　　降低企业经营成本。严格执行支持民营经济高质量发展的政策。坚决落实减税以及城市基础设施配套费、社保缴费等降费政策，清理规范商（协）会、中介收费，严厉查处乱收费，进一步降低企业合规成本、物流成本和各项要素成本。

　　推动市场主体成长。加强政策扶持，推动市场主体数量快速增长，争取突破150万户，其中企业超过55万户。持续推进“个转企、小升规、规改股、股上市”。鼓励民营企业建立现代企业制度，突出主业，打造核心竞争力。加大政府采购和政府购买服务对民营企业的支持力度。

　　构建亲清新型政商关系。落实领导干部联系企业制度。建立政商联系沟通服务平台，健全企业家参与涉企政策制定机制。充分发挥新组建的民营经济发展局在支持和服务民营企业发展中的牵头作用，充分发挥工商联和各类商（协）会的桥梁纽带作用。尊重企业市场主体地位，弘扬企业家精神，维护企业和企业家权益，让企业家有更多的荣誉感、自豪感、成就感。

　　（十一）提升区域经济发展能级。把准发展定位，补齐短板，拓展空间，加快形成各具特色、优势互补的区域协调发展格局。

　　提高重点功能区开发开放水平。推动开发区、高新区和保税港区体制放活、环境做优、产业做强、管理创新，打造高质量发展新引擎。创新管理和运营模式，集中精力抓经济管理、投资服务和规划建设，采取市场化方式解决高端人才紧缺、激励机制缺失等问题。扩大简政放权，将部分市级经济管理权限下放给功能区。完善功能区基础设施、生活设施配套，提升园区承载力，加快产城融合发展。引导产业项目向功能区定向集聚，实现特色化、高端化、规模化发展。

　　促进重点改革试验区创新发展。西海岸新区在服务落实国家重大战略中先行先试，当好改革开放的示范区、创新发展的排头兵、加快发展的驱动器。中国—上合组织地方经贸合作示范区开展好面向上合组织国家的物流、贸易、加工制造方面的深度合作，推动核心区实现突破性进展。蓝谷加快高技术产业项目建设，争创以海洋为特色的国家级高新区。胶东临空经济示范区启动消费中心项目建设，申建空港综合保税区。金融综合改革试验区加大金融、财富管理、金融科技等领域资源集聚和发展力度，打造新金融快速发展和财富管理高地。

　　促进区市经济蓬勃发展。区市要强基础挖优势，补短板避劣势，聚焦能够专业化、规模化发展的产业集群集中培育，打造一批重点产业集聚区，努力实现跨越式发展。有条件发展制造业的区市要牢固树立制造业立市（区）、制造业强市（区）、制造业富市（区）的理念，重点发展制造业以及配套服务业。中心城区要围绕服务业和总部经济、楼宇经济、楼宇工业等主题加快相关产业发展。区市要大力发扬比学赶帮超的精神，争先恐后加快发展；努力错位发展，避免同质化竞争。市级建立全域资源流动、企业搬迁合理的利益调整机制，健全加快发展的激励机制。市直主管部门要加强对各区市的调度和统筹，加强对薄弱部位的帮助和支持。

　　（十二）深入推进时尚美丽青岛建设。坚持世界眼光、国际标准、中国特色、高点定位，持续推进美丽青岛三年行动，推动城市规划科学化、建设精品化、管理精细化，提升城市功能品质。

　　高起点做好城市规划。持续推进“多规合一”，编制完成《青岛市2050远景发展战略》，加快国土空间规划和《青岛市城市总体规划（2018-2035年）》编制。发挥城乡规划综合管控和前瞻引领作用，维护规划的严肃性和权威性，切实做到“一张蓝图干到底”。深化总体城市设计，引导优化公共空间、街道景观、建筑立面，塑造城市特色，提升城市魅力。优化历史街区规划，促进老城区有机更新，传承历史文脉。

　　高标准实施城市建设。精心建设城市住宅、商业和公共设施，打造留得下、记得住的建筑精品。加快构建环湾一体的高快速路网体系。打通20条未贯通道路。完成4座水厂深度处理改造。扩建李村河、海泊河、麦岛污水处理厂。加快“世界一流城市配电网”建设。新建天然气管网200公里。统筹地上地下空间开发利用。推进地下综合管廊建设。完成国家海绵城市建设试点任务。新建改建公厕120座。新建改建绿地200公顷，改造口袋公园、景观节点50处。

　　高水平抓好城市管理。拿出绣花功夫，重在落细落小，提升城市管理和运行精细化便利化法治化水平。强化城市管理的标准规范体系建设，持续推进城市环境整治。落实街长制，提高网格化管理水平。加快美丽街区、美丽社区示范点建设，提升居民楼院和背街小巷环境品质。持续整治占道经营、露天烧烤、占绿毁林、私搭乱建和违法建设。全面推进生活垃圾分类,做好分类投放、分类收集、分类运输、分类处理。加强交通微循环改造，完善交通设施，增加停车泊位，推动智能交通系统建设城乡全覆盖。

　　（十三）加快建设宜业宜居宜游环境。对标先进，推进企业投资贸易便利化规范化、个人工作生活便利化品质化,营造“人人都是投资环境、事事关乎城市形象”的浓厚氛围。

　　努力营造高效的政务环境。聚焦“少、快、好”，持续深化“放管服”改革。积极扩大县域经济社会管理权限。再精简和下放一批审批事项，实现审批事项数量最少。深入推进“一次办好”改革，全面落实“3540”改革标准，整合市、区（市）政务服务事项进驻政务大厅，整合政府信息资源，全面推行“一窗受理”和“马上办、网上办、就近办、一次办”，实现审批速度最快。坚持真服务、服真务，为企业发展提供“保姆式”服务，当好企业发展的“店小二”，实现服务最好。

　　努力营造公正的法治环境。加强政府诚信建设，坚决说到做到，切实杜绝“新官不理旧账”。加强企业诚信和个人信用体系建设，建立信用“红黑名单”制度，完善守信联合激励和失信联合惩戒机制。坚持权力运行公开透明规范，努力减少不确定性。规范行政执法，确保公平公正。全面推行“双随机、一公开”市场监管方式。坚持运用市场规则和法律手段推进去产能、节能降耗等工作，制定明确和可预期的环保、质量、安全等标准。

　　努力营造便利的工作生活环境。突出便利化品质化，使居民个人工作生活更加舒适便捷。建设综合性政务服务自助办理平台，增加便民办理网点。推进城市信息化建设，构建覆盖民生服务、企业发展、城市管理的应用体系，提高服务智慧化水平。加强基层社区治理，推进公共服务进社区，完善生活性服务业布局，营造“15分钟生活圈”。积极发展城市慢行等便民交通设施体系。

　　（十四）着力增强文化软实力。深入挖掘历史文化名城内涵，弘扬科学精神、人文精神、艺术精神，繁荣发展文化事业，使城市文化特质更加彰显、精神品格更加鲜明、人文内涵更加丰厚。

　　深化文明城市创建。强化教育引导、实践养成、制度保障，把社会主义核心价值观融入社会发展各方面。挖掘利用好革命历史重要事件、重要人物和重要遗址，传承红色基因，深化爱国主义、集体主义和中国梦宣传教育。做好第六轮全国文明城市创建工作。实施公民道德建设工程。推进新时代文明实践中心建设试点。倡导移风易俗。开展志愿服务。繁荣发展哲学社会科学。打造新型高端智库。深化双拥共建，争创全国双拥模范城“九连冠”。加强国防教育和民兵预备役工作。加强普法宣传教育和法律援助工作。做好第四次经济普查工作。提升司法行政、民族宗教、侨务、台港澳、统计、档案、史志、仲裁、人防、气象、老龄、妇女儿童、青少年工作水平。

　　加强城市文化建设。倡导全民读书，让阅读和学习成为一种生活方式。挖掘文化底蕴，提升城市气质，塑造城市精神。实施全民科学素质行动计划，加强科普设施建设。启动扩建市博物馆。加快万国建筑博览区及中心馆建设，推进老城区申报世界文化遗产。发挥东方影都带动作用，繁荣发展影视文化。实施文化惠民工程，办好市民文化艺术节，推动公益演出、文化志愿服务走基层。深化“东亚文化之都”和“一带一路”文化交流。完善文艺精品奖励扶持机制，推出更多讴歌新时代的精品力作。办好中国（青岛）影博会、国际版权交易会等活动，争办国际青年电影节。

　　（十五）提高保障和改善民生水平。坚持把人民对美好生活的向往作为奋斗目标，不断加强民生保障，大力发展社会事业，让改革发展成果更多更公平惠及全市人民。

　　增加就业和居民收入。把稳就业摆在突出位置，落实就业优先战略和积极就业政策，实现城镇新增就业30万人以上。坚持以创业促就业，政策性扶持创业1.5万人。实施高校毕业生就业促进计划。做好退役军人安置就业和困难帮扶等工作，维护退役军人合法权益。抓好就业困难人员、贫困人口就业，实现就业1.5万人。调整最低工资标准和工资指导线。改革国有企业工资决定机制。支持科研人员通过科技成果转化获得合理收入。严格落实提高个人所得税起征点和专项附加扣除政策。

　　推动教育现代化发展。加快以学生德智体美劳全面发展为目标的教育改革。推进学前教育公益普惠发展，新建、改扩建标准化幼儿园60所。建设中小学标准化食堂100所。开展小学生课后校内托管基本服务，实施幸福家庭教育辅导服务工程。推动义务教育优质均衡发展。加大普通高中新建改建力度。筹建青岛工程职业学院，加快幼师新校区建设，推进职业教育调结构、上层次、提质量。支持驻青大学高水平发展，推进哈尔滨工程大学创新发展基地、北京航空航天大学科教新城、山东大学青岛校区二期工程建设。支持和规范社会力量兴办教育。办好继续教育、网络教育。全面深化新时代教师队伍建设改革。

　　发展卫生健康和体育事业。坚持以促健康、转模式、强基层为着力点，推动以治病为中心向以人民健康为中心转变，为人民群众提供全方位全周期健康服务。深化医药卫生体制改革，构建优质高效医疗卫生服务体系。做实家庭医生签约服务。加快基层卫生机构标准化建设。推进各重点医疗项目建设，新建20个院前急救站。实施振兴国医行动，建设40个精品国医馆。深化“互联网+医疗健康”，开展就医诊疗、结算支付、远程医疗等十大便民惠民服务。强化专项整治，建设“食安青岛”。开展全民健身活动，建设笼式足球场等健身场地100处，推动形成15分钟健身圈。办好2019世界休闲体育大会、“远东杯”国际帆船拉力赛、青岛马拉松等赛事，筹备第十四届全国学生运动会。

　　提高社会保障水平。深入推进全民参保计划。统筹调整离退休人员养老金、工伤人员伤残津贴标准。健全“全人全责”长期护理保险制度。实施全民补充医疗保险制度。扩大医疗保险异地联网结算定点机构范围。完善社会救助体系，加大临时救助力度。保障好城乡困难居民生活。加强残疾人康复服务，完善残疾人教育、就业扶持政策。促进人口均衡发展和健康老龄化。优化养老服务政策，建立困难失能老人养老服务补贴制度。健全农村留守儿童和妇女、老人关爱服务体系。支持公益慈善和红十字事业发展。

　　完善住房市场体系和保障体系。制定全市住房发展规划。按照国家统一部署搞好“一城一策”试点，加快建立促进房地产市场平稳健康发展长效机制。培育和发展住房租赁市场，开展集体建设用地建设租赁住房试点，支持企业利用自有土地依法合规建设公共租赁住房。探索共有产权住房制度。实施人才安居工程，新开工建设人才公寓100万平方米。实施住房保障6000套（户）。启动棚户区改造2.8万套（户）。改造农村危房489户。

　　抓好安全稳定工作。严格落实安全生产责任制，全面推行风险分级防控和隐患排查治理双重预防体系，坚决防范和遏制重特大事故发生。切实抓好森林防火工作。推进防灾减灾救灾能力建设，全面提升突发事件的预警、处置和救援能力。做好信访、稳定工作，加强人民调解组织和机制建设。提升社会治安综合治理水平，强化反恐、禁毒工作，深化打击治理电信网络新型违法犯罪，推动扫黑除恶专项斗争向纵深发展。

　　四、加强政府自身建设

　　始终牢记全心全意为人民服务的根本宗旨，深入落实全面从严治党要求，加快转变政府职能，全面提升履职水平，努力建设人民满意的服务型政府。

　　（一）加强政治建设。自觉用习近平新时代中国特色社会主义思想武装头脑、指导实践、推动工作。树牢“四个意识”，坚定“四个自信”，坚决做到“两个维护”，在政治立场、政治方向、政治原则、政治道路上同以习近平同志为核心的党中央保持高度一致。坚定坚决、不折不扣贯彻党中央决策部署和省委、市委工作要求，确保政令畅通、令行禁止。严守政治纪律和政治规矩，在重大原则问题和大是大非面前，做到立场坚定、旗帜鲜明。以高度的政治觉悟和强烈的责任担当抓好中央巡视整改。

　　（二）严格依法行政。加强法治政府建设，恪守宪法法律，切实按照法定职责用权，把政府工作全面纳入法治轨道。落实行政执法公示、执法全过程记录、重大执法决定法制审核制度，严格规范公正文明执法。认真执行市人大及其常委会的决议、决定，自觉接受市人大及其常委会法律监督、工作监督和市政协民主监督。做好建议提案办理工作。认真听取各民主党派、工商联、无党派人士和人民团体意见。落实政府法律顾问制度和公职律师制度。加强政府协商，坚持科学、民主、依法决策，凡涉及公众利益的重大事项，认真听取各方意见。深化政务公开，推进重大建设项目、公共资源配置、社会公益事业建设领域政府信息公开。加快政务服务热线整合，完善政务舆情回应机制。坚持开展“向市民报告、听市民意见、请市民评议”等系列活动。

　　（三）强化务实勤政。深入开展“工作落实年”活动，树立狠抓落实的工作导向，说实话、谋实事、出实招、求实效，以实干实绩取信于民。持之以恒推进思想再解放、观念再变革，持续在“六破六树”上下功夫，始终保持创新、求变、进取的活力。力戒形式主义、官僚主义，紧盯不敬畏、不在乎、喊口号、装样子的问题，拿出有效管用的措施坚决整治。认真践行群众路线，大兴调查研究之风，切实提高调研实效。深入贯彻激励广大干部新时代新担当新作为的意见，大力倡导敢担事、愿做事、能干事的精神，旗帜鲜明给积极干事者撑腰鼓劲，对庸政懒政者严肃问责。各级政府工作人员特别是领导干部要争当新时代泰山“挑山工”，勤政尽责、务实戒虚，扑下身子、沉到一线，推动各项工作落细落小落地落实。

　　（四）坚持廉洁从政。扛牢扛实全面从严治党政治责任，加强政府系统廉政建设。坚决落实中央八项规定精神和省、市委实施办法，时刻防范“四风”隐形变异新动向。树立过紧日子的思想，严格压缩一般性支出。加强公共资金、公共资源交易等廉洁风险突出的重点领域监管，坚决整治群众身边腐败和作风问题。主动接受纪检监察监督，支持审计机关依法依规履行职责。严格执行廉洁从政各项规定，做到严明责任不含糊、严抓纪律不放松、严管队伍不懈怠。

　　各位代表！新时代需要新担当,新使命需要新作为。让我们更加紧密地团结在以习近平同志为核心的党中央周围，高举习近平新时代中国特色社会主义思想伟大旗帜，在中共青岛市委的坚强领导下，不忘初心、牢记使命，凝心聚力、锐意进取，坚持把创新作为引领发展的第一动力，奋力把青岛建设得更加富有活力、更加时尚美丽、更加独具魅力，为实现在社会主义现代化新征程中率先走在前列努力奋斗，以优异成绩庆祝新中国成立70周年！
